# Efficacy of four commercially available heartworm preventive products against the JYD-34 laboratory strain of *Dirofilaria immitis*

**DOI:** 10.1186/s13071-016-1476-7

**Published:** 2016-04-05

**Authors:** Byron L. Blagburn, Robert G. Arther, Allen R. Dillon, Jamie M. Butler, Joy V. Bowles, Cristiano von Simson, Robert Zolynas

**Affiliations:** Department of Pathobiology, College of Veterinary Medicine, Auburn University, Auburn, AL USA; Department of Clinical Sciences, College of Veterinary Medicine, Auburn University, Auburn, AL USA; Clinical Development, Bayer HealthCare, Animal Health Division, Shawnee, KS USA

**Keywords:** *Dirofilaria immitis*, Heartworm, Canine, Prevention, Macrocyclic lactone, Efficacy, JYD-34, Resistance

## Abstract

**Background:**

Heartworm disease in dogs can be severe and life threatening. Resistance to available heartworm preventives was considered among potential causes of increased reports of failed heartworm prevention in dogs. The objective of the present study was to compare the efficacy of four commercially available heartworm disease preventives against the JYD-34 strain of *D. immitis.*

**Methods:**

Forty laboratory-reared dogs approximately 6 months old were used. Each dog was infected with fifty, third-stage heartworm larvae on study day (SD) -30. On SD-1, the dogs were randomized to five groups of eight dogs each. On SD-0, dogs in groups 1–4 were treated as follows: Group 1: ivermectin/pyrantel pamoate chewable tablets; Group 2: milbemycin oxime/spinosad tablets; Group 3: selamectin topical solution; and Group 4: imidacloprid/moxidectin topical solution. Dogs in Group 5 were not treated and served as controls. The dogs were treated according to their current body weights and labelled dose banding for each product. Groups 1, 2, and 3 were retreated with their respective products and current body weights on SD 31 and 60. On SDs 124–126 the dogs were euthanized and necropsied for recovery of adult heartworms*.*

**Results:**

Adult heartworms were recovered at necropsy from each of the dogs in the control group (13–32 worms/dog, geometric mean (GM) = 18.4 worms/dog). Adult heartworms and/or worm fragments were also recovered from each of the dogs treated with ivermectin/pyrantel pamoate, milbemycin oxime/spinosad or selamectin. Geometric means of worms recovered from dogs in each of these groups were 13.1, 8.8, and 13.1, resulting in efficacies compared to controls of 29.0, 52.2, and 28.8 %, respectively. All dogs in Group 4 (imidacloprid/moxidectin) were free of adult heartworms (100 % efficacy).

**Conclusions:**

The combination of imidacloprid/moxidectin was 100 % effective in this study in preventing development of JYD-34 laboratory strain of *D. immitis* in dogs following a single treatment, while three monthlytreatments of the three other commercial products provided less than 100 % efficacy. The high efficacy achieved with imidacloprid/moxidectin was likely due to the unique pharmacokinetic properties of the topical formulation delivering greater and sustained drug concentrations necessary to prevent development of *D. immitis* larvae.

## Background

Canine heartworm disease (CHD) can be a severe and life threatening condition [1, 2], which is normally prevented by prescribed treatments with macrocyclic lactone preventives including ivermectin, milbemycin oxime, moxidectin and selamectin. Resistance to available heartworm preventives was previously considered among potential causes of increased reports of failed heartworm prevention in dogs [3]. Prevention failures have also been attributed to the interplay of factors in the heartworm life-cycle, erratic treatment compliance, as well as diagnostic, prevention and therapeutic variables [4–7]. However, recent research has confirmed that certain heartworm isolates possess reduced susceptibility to macrocyclic lactone preventives [5, 8–10]. Prior research also indicates that efficacy of heartworm disease preventives may depend on the active ingredient and formulation as well as the treatment regimen [11–13]. Specific testing procedures must be used when evaluating heartworm preventives (http://www.fda.gov/downloads/animalveterinary/guidancecomGuidanceforIndustry/guidanceforindustry/ucm052417.pdf; http://www.fda.gov/downloads/animalveterinary/guidancecomplianceenforcement/guidanceforindustry/ucm052417.pdf; http://www.fda.gov/downloads/AnimalVeterinary/GuidanceComplianceEnforcement/GuidanceforIndustry/UCM052652.pdf.

Studies must be performed using recently characterized *D. immitis* strains with specified timing of treatment relative to experimental infection. The United States Food and Drug Administration Center for Veterinary Medicine requires 100 % efficacy for laboratory studies supporting the approval of heartworm disease preventive products. In a recent study, the JYD-34 laboratory strain of *D. immitis* (TRS Laboratories Inc., Athens, GA, USA) was used to test the performance of a new anthelmintic. Data indicated that milbemycin oxime was not 100 % effective against the JYD-34 heartworm strain at historically effective doses [14]. Therefore, the efficacy of other macrolide preventives against the JYD-34 heartworm strain was of interest.

Advantage Multi® [10 % imidacloprid/2.5 % moxidectin] Topical Solution for Dogs, is a topical formulation approved for prevention of heartworm disease, treatment of circulating microfilariae, treatment of flea infestations, and treatment and control of sarcoptic mange and intestinal nematodes One topical treatment, administered at the minimal label dose (0.1 ml/kg) at 30–45 days after experimental infection with *D. immitis*, resulted in 100 % efficacy [15]. Because of its lipophilic chemistry, moxidectin reaches high serum concentrations, broad tissue distribution and is gradually eliminated from the treated host. Moxidectin (Advantage Multi® for Dogs) reached a mean serum concentration of 18.1 mcg/L after one dosing with mean level of 10.5 mcg/L maintained for 28 days [16]. Based on these properties, we postulated that moxidectin in this formulation could provide a high level of efficacy against the JYD-34 strain of *D. immitis*. This study was conducted to compare the efficacy of Advantage Multi® for Dogs against JYD-34 following one treatment to the efficacy of three other heartworm preventive products administered three times at monthly intervals.

## Methods

This study was conducted as a controlled, blinded, laboratory efficacy study in accordance with current Good Clinical Practice^a^ and VICH anthelmintic guidelines (http://www.fda.gov/downloads/animalveterinary/guidancecomplianceenforcement/guidanceforindustry/ucm052417.pdf; http://www.fda.gov/downloads/AnimalVeterinary/GuidanceComplianceEnforcement/GuidanceforIndustry/UCM052652.pdf.

The study was reviewed and approved by the Auburn University Institutional Animal Care and Use Committee (PRN 2012–2040). Forty-three Beagle dogs, 6–7 months of age, were obtained from a commercial source (Ridglan Farms Inc. PO Box 318, Mt. Horeb, Wisconsin USA 53572). All dogs were allowed to acclimate to housing environments for 14 days. Housing of dogs was in indoor kennels within rooms equipped with automatic temperature controls. Each dog was fed a standard ration daily in quantities sufficient for growth and maintenance (Advanced Protocol High Density Canine Diet, PMI Nutrition, Int’l, LLC, Brentwood, MO 36144). The processed diet was determined to be free of any contaminants that could potentially interfere with the interpretation of study results. Each dog had access to municipal water provided *ad libitum via* individual valves. During the acclimation period, a physical examination was performed on each dog for the presence of existing conditions that could interfere with conduct of the study.

Each dog was infected with 50 third-stage heartworm larvae on study day (SD) -30. Larvae of JYD-34 were supplied by TRS Laboratories, Athens, Georgia, USA, and were harvested from infected mosquitoes (*Aedes aegypti*; Liverpool strain) immediately prior to inoculation. The JYD-34 *D. immitis* strain was originally isolated from a microfilariae-positive dog from Illinois, USA, in July, 2010. The dog had no previous history of treatment with an avermectin or milbemycin product. The strain was validated *via* experimental infection in April 2011 (Scott McCall, TRS Labs Inc., personal communication). The larvae harvested from mosquitoes for use in this study originated from microfilariae representing the first passage in a dog.

On SD-1, each dog was weighed. The three dogs at the extremes of the weight range (two largest; one smallest) were excluded from the study. The remaining 40 dogs were randomized to 5 groups of 8 dogs based on hierarchal weights. Treatment groups were then randomized as follows: Group 1: ivermectin/pyrantel pamoate (Heartgard® Plus Chewables for Dogs, Merial Ltd., Duluth, GA, USA); Group 2: milbemycin oxime/spinosad (Trifexis® Chewables for Dogs, Elanco Animal Health, Indianapolis, Indiana, USA); Group 3: selamectin (Revolution®, Zoetis Animal Health, Florham Park, New Jersey, USA); Group 4 : imidacloprid + moxidectin (Advantage Multi® for Dogs, Bayer HealthCare, Animal Health, Shawnee, Kansas, USA). Group 5 dogs served as non-treated controls.

The dogs assigned to Groups 1–4 were treated on SD-0 with the specific Investigational Veterinary Product (IVP) as described. Treatment dosages were based on body weights obtained on SD-1. The dosage of IVP for each dog was calculated based on each product’s package labelling. Food was withheld for 24 h prior to treatment for all dogs. For dogs treated with ivermectin/pyrantel and milbmycin/spinosad, tablets were placed on the back of the tongue to ensure reliable consumption of these products. For dogs treated with selamectin and imidacloprid/moxidectin, the appropriate volume of solution for each dog’s weight was administered topically as directed by label instructions. The non-treated control dogs were mock-treated to maintain proper masking of study participants. This was performed by bringing dogs into the treatment room and “pretending” to treat while other study participants waited outside the room. Dogs were allowed access to food immediately after treatment. Dogs in Groups 1, 2, and 3 were retreated with the respective IVPs as described above on SDs 31 and 60 based on body weights obtained on SDs 28 and 59, respectively. All dogs were observed for any adverse event or abnormal finding at selected intervals after treatment and at least once daily thereafter.

Plasma samples from each dog were examined on SD 94 for heartworm antigen (Dirochek® Canine Heartworm Antigen Test Kit, Zoetis Corporation, Florham Park, New Jersey, USA). Study animals were euthanized according to procedures currently used by the Auburn University Division of Laboratory Animal Health and necropsied on SDs 124–126 (154–156 days after inoculation with third stage *D. immitis* larvae) for recovery of adult heartworms. All dogs were examined for adult heartworms at necropsy as previously described [11].

Effects of the different treatments were determined by comparing heartworm numbers in dogs treated with the IVPs to those in non-treated dogs. Results are calculated and displayed as the number of positive dogs and geometric mean worm counts for dogs in each group. Geometric means were obtained by log-conversion of arithmetic mean counts of heartworms recovered from each dog. Mean counts were used to calculate efficacy as follows: Efficacy = Mean number of heartworms in control dogs – mean number of heartworms in treated dogs ÷ mean number of heartworms in control dogs × 100.

Due to the non-normal distribution of data, nonparametric analysis was utilized [11]. The Wilcoxon Rank Sum Test (two-tailed, *P* = 0.05) was used to assess treatment *vs* control effects. All statistical procedures were performed using SAS version 9.2 (SAS® Institute, Cary, North Carolina, USA).

## Results

No adverse events that were related to treatment with the IVPs were observed in this study. Specific information regarding treatment groups, dosages of IVP administered to each dog and recovery of adult heartworms are included Tables 1, 2, 3 and 4. Dosage ranges for dogs within each group were: Group 1: 6.4–11.9 μg/kg of ivermectin/5.4–10.0 mg/kg pyrantel pamoate; Group 2: 0.5–1.0 mg/kg of milbemycin oxime/30.8–60.1 mg/kg spinosad; Group 3: 6.6–12.9 mg/kg of selamectin; and Group 4: 2.8–6.7 mg/kg of moxidectin/11.3–26.7 mg/kg imidacloprid. Study day 31 dosages were: Group 1: 6.3–11.6 μg/kg ivermectin/5.3–9.7 mg/kg pyrantel pamoate; Group 2: 0.5–1.0 mg/kg of milbemycin oxime/32.3–57.6 mg/kg spinosad; and Group 3: 6.9–12.8 mg/kg of selamectin. Study day 60 dosages were: Group 1: 6.2–11.6 μg/kg ivermectin/5.2–9.8 mg/kg pyrantel pamoate; Group 2: 0.5–0.9 mg/kg milbemycin oxime/31.1–56.5 mg/kg spinosad; and Group 3: 9.8–13.1 mg/kg selamectin. Group 4 dogs were not retreated on days 31 and 60.

**Table 1:** Animal Information and SD 0 treatment dosages (Groups 1–5)

Treatment group	Animal identification	Sex	Body weight (lb)				
Ivermectin/Pyrantel				Tablet administered		Dose	
(Group 1)				Ivermectin (μg)	Pyrantel (mg)	Ivermectin (μg/kg)	Pyrantel (mg/kg)
	IDF-2	M	25.2	136	114	11.9	10.0
	UHH-2	M	23.3	68	57	6.4	5.4
	VTH-2	M	22.5	68	57	6.6	5.6
	IOF-2	M	21.5	68	57	7.0	5.8
	BFG-2	F	20.9	68	57	7.2	6.0
	PXC-2	F	20.8	68	57	7.2	6.0
	VVH-2	M	19.4	68	57	7.7	6.5
	GSC-2	F	19.3	68	57	7.8	6.5
Milbemycin/Spinosad				Tablet administered		Dose	
(Group 2)				Milbemycin (mg)	Spinosad (mg)	Milbemycin (mg/kg)	Spinosad (mg/kg)
	FCG-2	F	24.0	9.3	560	0.9	51.3
	IUE-2	F	23.0	9.3	560	0.9	53.6
	UDH-2	M	22.9	9.3	560	0.9	53.8
	VCG-2	F	21.5	9.3	560	1.0	57.3
	TPH-2	M	21.2	9.3	560	1.0	58.1
	XBH-2	M	20.5	9.3	560	1.0	60.1
	EZG-2	F	19.3	4.5	270	0.5	30.8
	KUH-2	M	17.6	4.5	270	0.6	33.8
Selamectin				Applicator tube		Dose	
(Group 3)				Selamectin (mg)		Selamectin (mg/kg)	
	OFF-2	M	24.7	120		10.7	
	VUH-2	M	23.2	120		11.4	
	EYE-2	F	21.5	120		12.3	
	UKG-2	F	21.3	120		12.4	
	AVG-2	F	20.9	120		12.6	
	GPG-2	F	20.4	120		12.9	
	GDE-2	F	18.6	60		7.1	
	ZXG-2	F	19.9	60		6.6	
Moxidectin/ Imidacloprid				Applicator tube		Dose	
(Group 4)				Moxidectin (mg)	Imidacloprid (mg)	Moxidectin (mg/kg)	Imidaclopridd (mg/kg)
	DIH-2	M	23.9	62.5	250	5.8	23.0
	UYH-2	M	23.3	62.5	250	5.9	23.6
	CGH-2	M	22.2	62.5	250	6.2	24.8
	FKC-2	F	21.5	62.5	250	6.4	25.6
	FJC-2	F	20.8	62.5	250	6.6	26.4
	VZG-2	F	20.6	62.5	250	6.7	26.7
	LAG-2	F	19.5	25.0	100	2.8	11.3
	THH-2	M	18.8	25.0	100	2.9	11.7
Non-treated control							
(Group 5)	UTH-2	M	24.6	NA	NA	NA	NA
	GKC-2	F	23.3	NA	NA	NA	NA
	TWH-2	M	21.8	NA	NA	NA	NA
	EDG-2	F	21.2	NA	NA	NA	NA
	RAF-2	M	21.0	NA	NA	NA	NA
	CRC-2	F	20.3	NA	NA	NA	NA
	TJH-2	M	20.2	NA	NA	NA	NA
	IXG-2	F	18.3	NA	NA	NA	NA

**Table 2:** Animal information and SD 31 treatment dosages (Groups 1–5)

Treatment group	Animal identification	Sex	Body weight (lb)				
Ivermectin/Pyrantel				Tablet administered		Dose	
(Group 1)				Ivermectin (μg)	Pyrantel (mg)	Ivermectin (μg/kg)	Pyrantel (mg/kg)
	IDF-2	M	28.9	136	114	10.4	8.7
	UHH-2	M	25.8	136	114	11.6	9.7
	VTH-2	M	23.7	68	57	6.3	5.3
	IOF-2	M	23.1	68	57	6.5	5.4
	BFG-2	F	23.0	68	57	6.5	5.5
	PXC-2	F	20.4	68	57	7.3	6.1
	VVH-2	M	21.2	68	57	7.1	5.9
	GSC-2	F	19.4	68	57	7.7	6.5
Milbemycin/Spinosad				Tablet administered		Dose	
(Group 2)				Milbemycin (mg)	Spinosad (mg)	Milbemycin (mg/kg)	Spinosad (mg/kg)
	FCG-2	F	23.5	9.3	560	0.9	52.4
	IUE-2	F	25.7	9.3	560	0.8	47.9
	UDH-2	M	23.4	9.3	560	0.9	52.6
	VCG-2	F	22.4	9.3	560	0.9	55.0
	TPH-2	M	26.7	9.3	560	0.8	46.1
	XBH-2	M	21.4	9.3	560	1.0	57.6
	EZG-2	F	22.5	9.3	560	0.9	54.8
	KUH-2	M	18.4	4.5	270	0.5	32.3
				Applicator tube		Dose	
Selamectin				Selamectin (mg)		Selamectin (mg/kg)	
(Group 3)	OFF-2	M	25.5	120		10.4	
	VUH-2	M	24.1	120		11.0	
	EYE-2	F	23.0	120		11.5	
	UKG-2	F	21.6	120		12.2	
	AVG-2	F	22.3	120		11.8	
	GPG-2	F	20.7	120		12.8	
	GDE-2	F	20.8	120		12.7	
	ZXG-2	F	19.0	60		6.9	
Moxidectin/ Imidacloprid				Applicator tube		Dose	
(Group 4)				Moxidectin (mg)	Imidacloprid (mg)	Moxidectin (mg/kg)	Imidaclopridd (mg/kg)
	DIH-2	M	24.4	NA	NA	NA	NA
	UYH-2	M	27.1	NA	NA	NA	NA
	CGH-2	M	23.6	NA	NA	NA	NA
	FKC-2	F	22.1	NA	NA	NA	NA
	FJC-2	F	20.8	NA	NA	NA	NA
	VZG-2	F	20.6	NA	NA	NA	NA
	LAG-2	F	20.7	NA	NA	NA	NA
	THH-2	M	19.1	NA	NA	NA	NA
Non-treated control							
(Group 5)	UTH-2	M	28.1	NA	NA	NA	NA
	GKC-2	F	25.2	NA	NA	NA	NA
	TWH-2	M	22.8	NA	NA	NA	NA
	EDG-2	F	21.5	NA	NA	NA	NA
	RAF-2	M	23.5	NA	NA	NA	NA
	CRC-2	F	21.6	NA	NA	NA	NA
	TJH-2	M	22.5	NA	NA	NA	NA
	IXG-2	F	19.5	NA	NA	NA	NA

**Table 3:** Animal information and SD 60 treatment dosages (Groups 1–5)

Treatment group	Animal identification	Sex	Body weight (lb)				
Ivermectin/Pyrantel				Tablet administered		Dose	
(Group 1)				Ivermectin (μg)	Pyrantel (mg)	Ivermectin (μg/kg)	Pyrantel (mg/kg)
	IDF-2	M	29.6	136	114	10.1	8.5
	UHH-2	M	25.7	136	114	11.6	9.8
	VTH-2	M	24.1	68	57	6.2	5.2
	IOF-2	M	23.7	68	57	6.3	5.3
	BFG-2	F	24.2	68	57	6.2	5.2
	PXC-2	F	21.6	68	57	6.9	5.8
	VVH-2	M	22.8	68	57	6.6	5.5
	GSC-2	F	20.2	68	57	7.4	6.2
Milbemycin/Spinosad				Tablet administered		Dose	
(Group 2)				Milbemycin (mg)	Spinosad (mg)	Milbemycin (mg/kg)	Spinosad (mg/kg)
	FCG-2	F	24.7	9.3	560	0.8	49.9
	IUE-2	F	26.4	9.3	560	0.8	46.7
	UDH-2	M	23.8	9.3	560	0.9	51.8
	VCG-2	F	22.5	9.3	560	0.9	54.8
	TPH-2	M	28.3	9.3	560	0.7	43.5
	XBH-2	M	21.8	9.3	560	0.9	56.5
	EZG-2	F	22.6	9.3	560	0.9	54.5
	KUH-2	M	19.1	4.5	270	0.5	31.1
Selamectin				Applicator tube		Dose	
(Group 3)				Selamectin (mg)		Selamectin (mg/kg)	
	OFF-2	M	27.1	120		9.8	
	VUH-2	M	26.3	120		10.0	
	EYE-2	F	24.6	120		10.7	
	UKG-2	F	20.7	120		12.8	
	AVG-2	F	22.6	120		11.7	
	GPG-2	F	21.8	120		12.1	
	GDE-2	F	21.8	120		12.1	
	ZXG-2	F	20.2	120		13.1	
Moxidectin/Imidacloprid				Applicator tube		Dose	
(Group 4)				Moxidectin (mg)	Imidacloprid (mg)	Moxidectin (mg/kg)	Imidaclopridd (mg/kg)
	DIH-2	M	26.1	NA	NA	NA	NA
	UYH-2	M	27.1	NA	NA	NA	NA
	CGH-2	M	24.8	NA	NA	NA	NA
	FKC-2	F	21.6	NA	NA	NA	NA
	FJC-2	F	22.2	NA	NA	NA	NA
	VZG-2	F	20.6	NA	NA	NA	NA
	LAG-2	F	20.1	NA	NA	NA	NA
	THH-2	M	20.3	NA	NA	NA	NA
Non-treated control							
(Group 5)	UTH-2	M	27.6	NA	NA	NA	NA
	GKC-2	F	25.7	NA	NA	NA	NA
	TWH-2	M	23.9	NA	NA	NA	NA
	EDG-2	F	23.2	NA	NA	NA	NA
	RAF-2	M	24.4	NA	NA	NA	NA
	CRC-2	F	21.4	NA	NA	NA	NA
	TJH-2	M	24.2	NA	NA	NA	NA
	IXG-2	F	20.7	NA	NA	NA	NA

**Table 4:** Adult *D. immitis* recovered at necropsy

Treatment group	Animal ID	Male *D. immitis*	Female *D. immitis*	Total *D. immitis*
Ivermectin/Pyrantel	IDF-2	2	3	5
(Group 1)	UHH-2	12	3	16 ^a^
	VTH-2	10	5	15
	IOF-2	12	2	14
	BFG-2	5	7	13 ^a^
	PXC-2	6	8	15 ^a^
	VVH-2	12	7	19
	GSC-2	5	8	13
Total				110
Milbemycin/Spinosad	FCG-2	6	1	7
(Group 2)	IUE-2	2	0	3 ^a^
	UDH-2	9	4	14 ^a^
	VCG-2	3	1	4
	TPH-2	6	8	15 ^a^
	XBH-2	7	4	12 ^a^
	EZG-2	4	3	8 ^a^
	KUH-2	11	7	18
Total				81
Selamectin	OFF-2	7	9	17 ^a^
(Group 3)	VUH-2	4	4	9 ^a^
	EYE-2	6	2	9 ^a^
	UKG-2	4	8	13 ^a^
	AVG-2	9	5	15 ^a^
	GPG-2	12	4	16
	ZXG-2	13	5	18
	GDE-2	7	3	11 ^a^
Total				108
Imidacloprid/Moxidectin	DIH-2	0	0	0
(Group 4)	UYH-2	0	0	0
	CGH-2	0	0	0
	FKC-2	0	0	0
	FJC-2	0	0	0
	VZG-2	0	0	0
	LAG-2	0	0	0
	THH-2	0	0	0
Total				0
Non-treated Control	UTH-2	5	11	17 ^a^
(Group 5)	GKC-2	7	7	15 ^a^
	TWH-2	9	9	18
	EDG-2	9	8	18 ^a^
	RAF-2	6	15	21
	CRC-2	11	7	18
	TJH-2	4	9	13
	IXG-2	19	13	32
Total				152

^a^Intact heartworms were enumerated by gender. If worm pieces/ fragments were also recovered, one additional worm was added to the count for that dog

Plasma samples collected from each dog on SD 94 were negative for heartworm antigen.

Adult heartworms recovered from dogs at necropsy included intact worms or fragments and were enumerated based on gender (Table 4). All intact heartworms were alive and moving when placed in warm saline. A total of 152 adult D*. immitis* (range of 13–32 worms/dog) were recovered from dogs in the non-treated control group. The numbers of heartworms in the control dogs was sufficient to satisfy the regulatory adequacy of infection requirements for study validity.

Three or more heartworms were present in each dog from Groups 1–3; ≥ 15 worms were recovered from two or more dogs in each of these three groups. No heartworms were recovered from the dogs that were treated with imidacloprid/moxidectin. Geometric worm counts, efficacy calculations, and *P*-values are displayed in Table 5. Percent efficacy for Groups 1–4 was 29.0, 52.2, 28.8 and 100 %, respectively. Only the milbemycin + spinosad (52.2 % efficacy) and imidacloprid + moxidectin (100 % efficacy) treatment groups exhibited significantly fewer adult heartworms than the control group (*Z* = -2.59, *P* = 0.020 and *Z* = -3.55, *P* = 0.003, respectively).

**Table 5:** Efficacy and summary statistics

Treatment group	N	No. of positive dogs	Geometric mean no. of worms^c^	Percent efficacy ^d^	Treated *vs* Non-treated *P*-value (*Z*-value)
Ivermectin/ Pyrantel ^a^ (Group 1)	8	8	13.1	29.0	0.069 (-1.96)
Milbemycin Oxime/ Spinosad^a^ (Group 2)	8	8	8.8	52.2	0.020 (-2.59)
Selamectin^a^ (Group 3)	8	8	13.1	28.8	0.046 (-2.18)
Imidacloprid/ Moxidectin^b^ (Group 4)	8	0	0.0	100.0	0.003 (-3.55)
Non-treated Control (Group 5)	8	8	18.4	NA	NA

^a^Administered as three treatments on SD 0, 31 and 60

^b^Administered once on SD 0

^c^Number of positive dogs/Number of dogs in treatment group × 100

^d^Geometric mean number of worms (non-treated)-geometric mean number of worms (treated)/geometric mean number of worms (non-treated) × 100

## Discussion

The majority of cases of presumed heartworm prevention failures appear to be due to a lack of understanding of the heartworm life-cycle, lack of compliance with the use of heartworm disease preventatives, infrequent heartworm testing, and interpretation of testing results [4]. However, resistance has been documented and a strategy for protecting the available preventives must be determined [9, 10, 17–20]. Recommendations that represent sensible strategies for prevention of the further spread of resistant heartworms include placement of dogs on preventive as early as product labels allow, aggressively assisting dog owners in avoiding purchase gaps, assurance that the amount of product dispensed is sufficient to protect all dogs in the family, assurance that dogs received the proper dosage of preventive and that the entire dose is given, elimination of existing infections by proper use of the approved adulticide, elimination of microfilariae with an approved product and/or an effective regimen, and testing dogs annually for both adult worms and microfilariae [4, 21, 22].

The JYD-34 strain of *D. immitis* used in this study was isolated in July 2010 from a dog in Illinois, USA, and validated as a successful passage to a recipient Beagle dog in April 2011. Although complete information is lacking, there is no information to indicate that JYD-34 received previous exposure to macrocyclic lactone (ML) preventives. Previous research on the MP3 strain of *D. immitis* indicates that resistant isolates occur in the field, and that selective pressure using preventive doses of ML preventives could result in further selection of resistant heartworm biotypes [5, 23]. At present, neither the prevalence nor the geographic distributions of resistant heartworms is known. However, ongoing research continues to identify useful genetic markers that may be helpful in determining such prevalence.

Results obtained in this study demonstrate that not all available heartworm preventives are effective against the JYD-34 strain of *D. immitis*, even when they are administered monthly for three consecutive months. The results support the need for continuing research on the detection, characterization, prevalence and distribution of susceptible and resistant heartworm isolates. Current heartworm preventives all utilize MLs as their active preventive agent. As demonstrated herein and previously, there are differences in the performance of available preventive products against resistant heartworms. However, it remains prudent to combine product selection strategies with other aspects of heartworm prevention discussed previously to preserve the efficacy of these agents.

Group 4 (imidacloprid/moxidectin) was the only treatment group in the current study in which 100 % efficacy against the development of adult heartworms was achieved. The higher ML dosage delivered with this formulation *vs* the other three products evaluated in this study, in conjunction with the pharmacokinetics of moxidectin in the topical formulation, provided drug levels necessary to prevent the development of JYD-34 larvae to the adult stage in these dogs. In prior research, milbemycin oxime given as multiple treatments was more effective than single treatments against *D. immitis* [12, 13, 24]. It is also noteworthy that heartworm prevention failures documented from the Mississippi Valley USA were in dogs that were on monthly year-round prevention [5, 17, 19]. Since many pet owners fail to administer heartworm prevention products compliantly, a heartworm preventive that is 100 % effective as a single treatment against susceptible and resistant heartworms is advisable.

## Conclusions

Results reported herein indicate that successful prevention of JYD-34 heartworm infections were not achieved with all IVPs*,* even when some treatments were applied 30, 61 and 90 days after heartworm infection. Imidacloprid/moxidectin (Advantage Multi® for Dogs, Bayer HealthCare, Animal Health, Shawnee, Kansas, USA) was 100 % effective against the JYD-34 heartworm strain, following a single treatment The observed differences with imidacloprid/moxidectin treatment compared to the other IVPs tested in this study were due to the higher moxidectin dose banding, high lipophilicity of moxidectin,and the unique topical formulation that provides sustained serum levels with extensive tissue distribution following a single topical treatment.

## References

[1] Bowman DD, Atkins CE (2009). Heartworm biology, treatment, and control. Vet Clin N Am/Small Anim Pract.

[2] McCall JW, Genchi C, Kramer LH, Guerrero J, Venco L (2007). Heartworm disease in animals and humans. Adv Parasitol.

[3] Hampshire VA (2005). Evaluation of efficacy of heartworm preventive products at the FDA. Vet Parasitol.

[4] Atkins CE, Murray MJ, Olavessen LJ, Burton KW, Marshall JW, Brooks CC (2014). Heartworm ‘lack of effectiveness’ claims in the Mississippi delta: Computerized analysis of owner compliance – 2004–2011. Vet Parasitol.

[5] Blagburn B, Dillon R, Prichard R, Geary T, Mount J, Land T, Butler J, Bourguinat C. Characterization of heartworm prevention failures in the central United States. In Proc 13th Triennial Heartworm Symp. 16–18 April 2010, Memphis, 27

[6] Patton S, Odoi A, Rohrbach BW. Survey of heartworm prevention practices among dog owners and trainers in North America. In Proc 13th Triennial Heartworm Symposium. 16*–*18 April 2010, Memphis, 26

[7] Rohrbach BW, Odoi A, Rohrbach BW. Survey to identify risk factors for failure to prevent heartworm infection in dogs. In Proc 13th Triennial Heartworm Symposium. 16*–*18 April 2010, Memphis, 26

[8] Bourguinat, C, Keller, K, Schenker, R, Blagburn, B, Prichard, R, Geary, T. Investigation of genetic changes in *Dirofilaria immitis* after the use of macrocyclic lactone heartworm preventives. In Proc 13th Triennial Heartworm Symposium. 16–18 April 2010, Memphis, 28

[9] Bowman D (2012). Heartworms, macrocyclic lactones, and the specter of resistance to prevention in the Unites States. Parasites Vectors.

[10] Bourguinat C, Lee A, Lizunda R, Blagburn B, Liotta J, Kraus M, Keller K, Epe C, Letourneau L, Kleinman C, Paterson T, Gomes E, Montoya-Alonso J, Smith H, Bhan A, Peregrine A, Carmichael J, Drake J, Schenker R, Kaminsky R, Bowman D, Geary T, Prichard R (2015). Macrocyclic lactone resistance in *Dirofilaria immitis*: Failure of heartworm preventives and investigation of genetic markers for resistance. Vet Parasitol.

[11] Blagburn B, Dillon R, Arther R, Butler J, Newton J (2011). Comparative efficacy of commercially available heartworm preventative products against the MP3 laboratory strain of *Dirofilaria immitis*. Vet Parasit.

[12] Snyder D, Wiseman L, Cruthers L, Slone R (2011). Ivermectin and milbemycin oxime in experimental adult heartworm (*Dirofilaria immitis*) infections of dogs. J Vet Intern Med.

[13] Snyder D, Wiseman S, Bowman D, McCall J, Reinemeyer C (2011). Assessment of the effectiveness of a combination product of spinosad and milbemycin oxime on the prophylaxis of canine heartworm infection. Vet Parasitol.

[14] Assessment Report for Nexgard Spectra (EMEA/V/C/003842/0000). Committee for Medicinal Products for Veterinary Use (CMVP). http://www.ema.europa.eu/docs/en_GB/document_library/EPAR_-_Public_assessment_report/veterinary/003842/WC500181963.pdf. Accessed 27 Aug 2015.

[15] Arther RG, Bowman DD, Slone RL, Travis LE (2005). Imidacloprid plus moxidectin topical solution for the prevention of heartworm disease (*Dirofilaria immitis*) in dogs. Parasitol Res.

[16] Beddies G. Serum pharmacokinetics of imidacloprid 10 % w/vmoxidectin w/v 2.5 % spot-on in dogs applied once per month for three consecutive monthly intervals. FOI Summary *-* Advantage Multi for Dogs 2006, NADA-141-251.

[17] Blagburn B, Carmichael J, Kaminsky R, Schenker R, Kaplan R, Moorhead A, Prichard R, Geary T, Bourguinat C, Malone J, Bowman D. Resistance and heartworm preventives: historical perspective and overview of research. In Proc 58th Annual Mtg Am Assoc Vet Parasitol. 20–23 July 2013, Chicago, IL, 39

[18] Bowman D, Lee A, Harrington L, Ledesma N, Kraus M, Liotta J, Blagburn B, Drake J, Carmichael J, Schenker R. Testing the efficacy of an injectable moxidectin formulation (ProHeart® 6) against a field isolate of canine heartworm. In Proc 58th Annual Mtg Am Assoc Vet Parasitol. 20–23 July 2013, Chicago, IL, 39

[19] Pulaski C, Malone J, Ward D, Klei, T, Pariaut R, Bourguinat C, Prichard R, Carmichael J, Schenker R. The establishment of macrocyclic lactone resistant *Dirofilaria immitis* isolates in experimentally infected laboratory dogs. In Proc 58th Annual Mtg Am Assoc Vet Parasitol. 20–23 July 2013, Chicago, IL, 39 10.1186/s13071-014-0494-6 PMC4228187 25376278

[20] Kaminsky R, Lizundia, R, Blagburn B, Bowman, D, Carmichael J, Schenker R, Epe C, Sager H. Efficacy studies in dogs demonstrate resistance of Dirofilaria against ivermectin and other macrocyclic lactones. In Proc 58th Annual Mtg Am Assoc Vet Parasitol, 20–23 July 2013, Chicago, IL, 39.

[21] Current canine guidelines for the diagnosis, prevention and management of heartworm (*Dirofilaria immitis*) infection in dogs (revised 2014). American Heartworm Society. http://heartwormsociety.org. Accessed 04 Aug 2015.

[22] Current advice on parasite control: Heartworm – canine heartworm (2013). Companion Animal Parasite Council. http://capcvet.org. Accessed 04 August 2014.

[23] Blagburn B, Bowles J, Loechel R, Carmichael J, Schenker R, Roycott L. Evidence of genetic selection following treatment of a heartworm-infected, microfilaremic dog with increasing dosages of ivermectin. In Proc 58th Annual Mtg Am Assoc Vet Parasitol. 20–23 July 2013, Chicago, IL. 39

[24] Grieve RB, Frank GR, Stewart VA, Parsons JC, Belasco JC, Hepler DI (1991). Chemoprophylactic effects of milbemycin oxime against larvae of *Dirofilaria immitis* during prepatent development. Am J Vet Res.

